# Impact of stride-coupled gaze shifts of walking blowflies on the neuronal representation of visual targets

**DOI:** 10.3389/fnbeh.2014.00307

**Published:** 2014-09-15

**Authors:** Daniel Kress, Martin Egelhaaf

**Affiliations:** ^1^Department of Neurobiology, Bielefeld UniversityBielefeld, Germany; ^2^CITEC Center of Excellence Cognitive Interaction Technology, Bielefeld UniversityBielefeld, Germany

**Keywords:** spatial vision, blowfly, head movements, goal-directed, walking, fixation

## Abstract

During locomotion animals rely heavily on visual cues gained from the environment to guide their behavior. Examples are basic behaviors like collision avoidance or the approach to a goal. The saccadic gaze strategy of flying flies, which separates translational from rotational phases of locomotion, has been suggested to facilitate the extraction of environmental information, because only image flow evoked by translational self-motion contains relevant distance information about the surrounding world. In contrast to the translational phases of flight during which gaze direction is kept largely constant, walking flies experience continuous rotational image flow that is coupled to their stride-cycle. The consequences of these self-produced image shifts for the extraction of environmental information are still unclear. To assess the impact of stride-coupled image shifts on visual information processing, we performed electrophysiological recordings from the HSE cell, a motion sensitive wide-field neuron in the blowfly visual system. This cell has been concluded to play a key role in mediating optomotor behavior, self-motion estimation and spatial information processing. We used visual stimuli that were based on the visual input experienced by walking blowflies while approaching a black vertical bar. The response of HSE to these stimuli was dominated by periodic membrane potential fluctuations evoked by stride-coupled image shifts. Nevertheless, during the approach the cell’s response contained information about the bar and its background. The response components evoked by the bar were larger than the responses to its background, especially during the last phase of the approach. However, as revealed by targeted modifications of the visual input during walking, the extraction of distance information on the basis of HSE responses is much impaired by stride-coupled retinal image shifts. Possible mechanisms that may cope with these stride-coupled responses are discussed.

## Introduction

Gathering information about the external world during self-motion is a fundamental challenge for visually guided animals. Think, for example, of a task where an object needs to be detected and fixated during locomotion, before it can be successfully approached. In such a situation the retinal image displacements are not only affected by potential motion of the object, but also by the way the animal moves itself and by its ability to stabilize its gaze. Information about the spatial layout of the environment, in particular about objects, can be extracted most parsimoniously during translational self-motion, as here, in contrast to rotational self-motion, distance information is immediately reflected in the retinal image flow (Gibson, 1950). Flying insects have been shown to make use of this geometrical principle by employing a flight and gaze strategy that separates phases of brief saccade-like rotations from intersaccadic phases, which contain almost pure translations (Land, 1973; Schilstra and van Hateren, 1999; van Hateren and Schilstra, 1999; Boeddeker et al., 2010; Braun et al., 2010, 2012; Geurten et al., 2010; review: Egelhaaf et al., 2012).

In studies in which we analyzed the gaze behavior in a goal-directed paradigm, we found that freely walking flies have a different gaze behavior than flying ones. During walking they hardly ever show purely translational locomotion phases. Rather, blowflies perform relatively large periodic rotations of their body around all of its axes, which are caused by their walking apparatus. While stride-induced body rotations around the roll and pitch axes are compensated by counter-rotations of the head, body turns around the yaw axis are in general followed by the head (Kress and Egelhaaf, 2012). Hence, while approaching an object, walking flies experience relatively fast rotational image motion with velocities of up to ±170°/s around the yaw axis and amplitudes of up to 4° (Kress and Egelhaaf, 2014). These rotations are modulated at the stride frequency of about 12 Hz even during otherwise straight walking phases. Similar kinematic results for walking flies have been obtained in a pionieering study by Horn and Mittag (1980). Even when solving demanding visual tasks like the fixation of a moving object or the fixation of a stationary object in front of a moving background, stride-coupled gaze shifts were not compensated, indicating that these shifts are an inherent feature of walking (Kress and Egelhaaf, 2014). This finding was surprising, because flies have well established visually controlled compensation mechanisms (e.g., Götz and Wenking, 1973; Götz, 1975; Srinivasan, 1977; Hengstenberg, 1984), which operate within the dynamical range of stride-induced image displacements, and blowflies were shown to apply them during tethered walking and flight (compensation of body roll by head movements: Hengstenberg, 1993; Schwyn et al., 2011).

The consequences of these stride-coupled gaze shifts for the performance of flies in visual object-directed behavior have not been addressed systematically, so far, as most behavioral studies dealing with visual control mechanism were not able to resolve stride-induced body movements or worked with tethered animals precluding stride-induced body rotations (Götz and Wenking, 1973; Reichardt, 1973; Götz, 1975; Reichardt and Poggio, 1976; Virsik and Reichardt, 1976; Wehrhahn and Hausen, 1980; Egelhaaf, 1987; Kimmerle et al., 2000; Aptekar et al., 2012; Bahl et al., 2013; Fox et al., 2014; Fox and Frye, 2014). Therefore, the impact of stride-induced retinal image shifts on visual information processing is still unclear. Because walking flies do not separate rotational from translational phases as they do in flight (see above), spatial vision appears to be more challenging during walking than during flight. Still, there might be a computationally cheap visual mechanism to obtain rotation-independent translatory image flow that contains object and distance information: by subtracting the retinal velocities of the left and the right edge of an approached object, its expansion velocity and, thus, proximity information can be estimated, irrespective of superimposed stride-coupled rotational image shifts. These rotational shifts are the same for the two edges of the object and, thus, can be eliminated by a subtractive mechanism (Kress and Egelhaaf, 2014). Other possibilities are non visual mechanisms, such as mechanosensory feedback from the walking machinery or an efference copy originating in the motor control system and generating a signal proportional to the stride-coupled rotational image shifts.

Here, we investigate how the retinal image flow experienced by freely walking flies in an object fixation task is represented at the output level of the fly’s visual motion pathway. Flies have a class of wide-field motion sensitive visual interneurons that are known to be key players in motion information processing. These Lobula plate tangential cells (LPTCs) are part of the fly’s third visual neuropile and integrate retinotopically organized local motion inputs (e.g., Egelhaaf, 2006; Borst et al., 2010; Maisak et al., 2013; Takemura et al., 2013). The resulting motion selectivity within their large receptive fields appears to match the image-motion evoked by self-motion of the animal through its environment (Krapp et al., 1998; Franz and Krapp, 2000; Krapp et al., 2001). Therefore, LPTCs are thought to act as self-motion detectors at least for motion velocities within their ideal working range (Karmeier et al., 2006). This property as well as the finding that they directly project to the head motor system, suggest that LPTCs could play a fundamental role in the above described gaze compensation (Strausfeld and Seyan, 1985; Milde and Strausfeld, 1986; Huston and Krapp, 2008, 2009; Haag et al., 2010; Wertz et al., 2012). Moreover, LPTCs sensitive to horizontal motion have been concluded to extract distance information from the image motion caused by translational self-motion during flight (Kern et al., 2005; Karmeier et al., 2006; Liang et al., 2008, 2012; review: Egelhaaf et al., 2012).

By recording the activity of HSE cells, a specific LPTC, during stimulation with image motion as perceived by freely walking flies in a goal-directed paradigm, we addressed three open questions: (i) How strong is the stride-induced response component in relation to the overall responses of HSE cells?; (ii) How do stride-induced gaze shifts interfere with the representation of external information in HSE cells?; and (iii) To what extent do HSE cell responses reliably reflect the retinal edge velocities? A pronounced edge velocity response component would be required if the consequences of rotational stride-coupled image flow are to be eliminated by subtracting the edge velocities of the object (see above). To address these questions we presented in our electrophysiological experiments the image flow as seen during object-induced behavior, as well as modified versions of it.

## Materials and methods

### Animals and electrophysiology

We dissected 1–3-day-old female blowflies, *Calliphora vicina* (taken from the laboratory stock) as described previously (Dürr and Egelhaaf, 1999) with the exception that we did not remove the gut and the heart. Immobilized animals were aligned according to the pseudopupil orientation (Franceschini, 1972) and fixed on a custom built holder, which was then placed in the center of the current version of our high-speed, panoramic LED arena, FliMax (see below). During electrophysiological recordings, the temperature close to the animal ranged between 26–32°C. Neuronal activities of HSE-cells in the right brain hemisphere were recorded intracellularly with sharp borosilicate electrodes (G100TF-4, Warner Instruments, Hamden, CT, USA) pulled on a Brown-Flaming puller (P1000, Sutter Instruments, Novato, CA, USA). Electrodes were filled with 1 M KCl and had resistances of 20–55 MΩ. To prevent the brain from desiccation, we used Ringer solution (Kurtz et al., 2000), manually applied via the indifferent electrode. The signal was amplified and low-pass filtered (cutoff frequency: 2.4 kHz) by a custom built amplifier (TK 88, Max-Planck-Institute for Biological Cybernetics, Tübingen, Germany) and thereafter digitized at a rate of 8.192 kHz (DT3001 l/0-card, Data Translation, Marlboro, MA, USA). The MATLAB data acquisition toolbox (The MathWorks Inc., Natick, MA, USA) was used to store the recorded data for offline analysis.

### Visual stimulus generation and presentation

**Figure 1:**
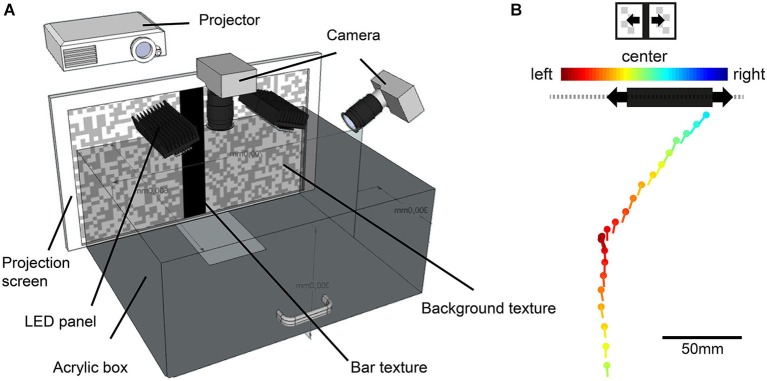
**Illustration of the behavioral experiments. (A)** Walking arena made of infrared-transparent acrylic. Walking blowflies were recorded with two high-speed cameras while approaching a black vertical bar projected onto the a projection screen. **(B)** Example trace of a fly approaching a horizontaly moving bar. The fly’s head position (dots) and head yaw orientation (lines) are shown for 100 ms intervals. Color code of dot and lines indicates the position of the moving bar’s center. Warm colors indicate a position to the left, cold colors a position to the right of its central position. The black bar position represents the position of the bar at the end of the approach. The gray dashed line illustrates the textured background.

Visual stimuli were based on what freely walking flies had previously seen in a visual orientation paradigm. The behavioral data were obtained in a another study for which we developed a precise head tracking technique allowing fly gaze estimation along the walking trajectories in an arena (Kress and Egelhaaf, 2014). In short, walking flies were monitored by two infrared-sensitive cameras (CR 600, Optronis GmbH, Kehl, Germany), equipped with DG MACRO 24–70 mm lenses (SIGMA GmbH, Roedermark, Germany; resolution: 1280 × 1024 pixel) at 200 frames per second. The walking arena consisted of an infrared-transparent acrylic box (70 × 60 × 30 cm, width × length × height) with a rear projection screen (Studio®, Gerriets GmbH, Umkirch, Germany) as front wall. It was placed in a dark room (Figure 1A). The left side wall of the arena was covered with white cardboard containing a hole for the side camera. The opposite side wall was equally textured including a dummy camera hole to keep the arena appearance symmetrical. The arena floor was covered with black cardboard. The acrylic walls were specially coated to allow only light of wavelengths larger than 700 nm to pass the walls (LUXACRYL-IR, ttv gmbh, Geretsried, Germany). As light sources we used panels of IR LEDs with a peak emission of either λ = 890 nm or λ = 850 nm. The panels thus emitted light at wavelengths far beyond the sensitive range of fly photoreceptors (Hardie, 1979). As a consequence, the projection screen displaying the visual stimulus was the only perceivable light source for the tested flies.

During the behavioral experiments, the fly approached a high-contrast black vertical bar in front of a random textured background, projected onto the front wall of a box-shaped arena (Figure 1B). The bar had a size of 5.8 cm × 30 cm (width × height), corresponding to 16° × 56° at a viewing distance of 20 cm. The background consisted of a random pattern of gray and white squares of 1 cm edge length and an angular extent of 2.9° when seen from 20 cm distance (Figure 1A). Object-induced approaches of the bar were tested under three conditions: (1) both bar and background were stationary; (2) the bar oscillated in front of the stationary background (Figure 1B); and (3) the bar was stationary and the background oscillated. The bar or the background oscillated at 5 cm s^−1^ and a frequency of 0.25 Hz around the center of the frontal arena wall, corresponding to an angular velocity of 14° s^−1^ at a distance of 20 cm.

For reconstruction of ego-perspective movies that were used as stimuli in our electrophysiological analysis, we selected three walks, one for each stimulus condition, from our database gathered in another study (Kress and Egelhaaf, 2014). We selected the walking traces according the criterion that the performance in fixating the bar under the three conditions was close to the average performance of the entire database. The most important criteria were: (1) The walking flies performed stride coupled head rotations that reflected the amplitude and frequency of the average across flies; (2) The flies had at least one stop phase during the approach; (3) Their walking speed was close to the average walking speed across flies; (4) They approached the bar at its edge; (5) They performed yaw saccades either after stop phases or during continuous walking or both; and (6) the directedness of their approach was similar to the average across flies. For technical reasons we could not use the same individual flies for behavioral and electrophysiological experiments. The estimation of the position and orientation of the fly’s head in the pairs of movie frames was accomplished by automatic tracking of white marker points attached to it. By calculating the vector orientation between marker points and comparing it to the orientation of a reference line through the setup, we obtained the head’s yaw orientation relative to the center of the projection screen (for details see “2D method” described in Kress and Egelhaaf, 2012). To reconstruct the visual input encountered by the freely walking flies during the approach to the bar, we combined the fly’s head position and orientation (Figure 1B), the respective position of the bar and the background on the projection screen as well as the interior appearance of the walking arena (Figure 1A) in a computer model created in Open Inventor.^1^ These data were used to render the ego-perspective stimulus movies presented on our panoramic LED arena, FliMax (for more information about the rendering procedure see: Lindemann et al., 2003; Geurten et al., 2012; Liang et al., 2012). The stimuli were either shaped exclusively by self-induced image shifts (stationary stimulus condition) or a combination of self-produced and external motion cues of the bar (moving bar condition) or of the textured background (moving background condition). The Michelson-contrast between the bar and the white parts of the textured background recorded in FliMax was *c* = 0.81 (bar luminance: 2200 cd m^−2^; white background patches luminance: 22,000 cd m^−2^, recorded with a luminance meter: Konica Minolta Sensing LS-100, Osaka, Japan). Contrast between the bar and gray parts of the background resulted in *c* = 0.69 (gray background patches luminance: 12,000 cd m^−2^). The black floor had a luminance of 350 cd m^−2^. Stimulus movie length was similar for the different conditions, ranging from 2.34–2.48 s.

**Figure 2:**
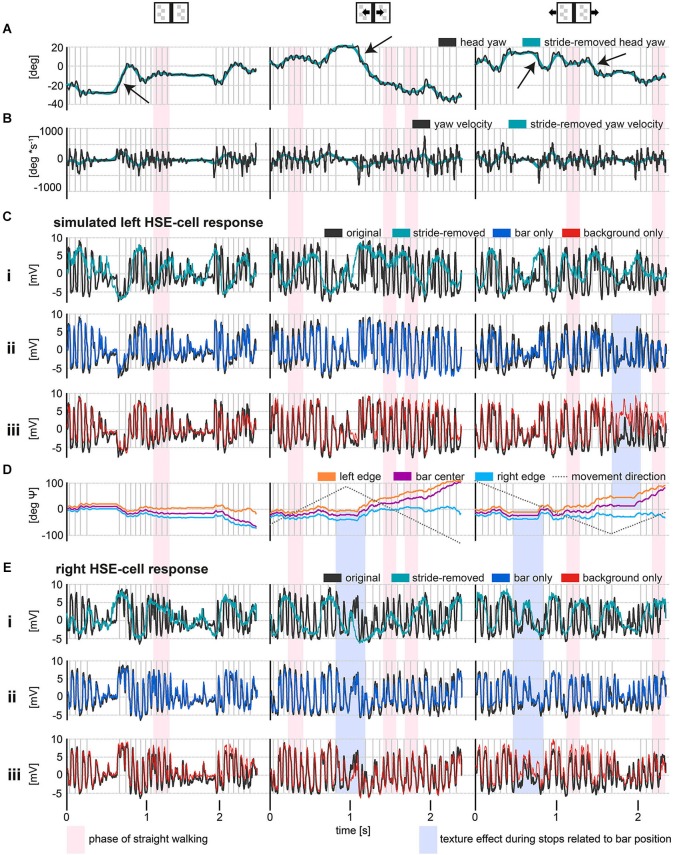
**HSE responses during stimulation with reconstructed optic flow, as experienced by freely walking flies approaching a goal**. The external texture cues were either stationary or moving (see insets above data plots). The gray vertical lines indicate the touchdown of the left midleg. Red shaded areas indicate phases of relatively straight walking. Blue shaded areas mark the recorded membrane potentials of the right and the simulated left HSE-cell during stop phases, in which retinal image motion is evoked only by the motion of external textures and related to bar positon. **(A)** Original and stride-removed yaw orientation of the head relative to a horizontal axis in the walking area. Positive values indicate a leftward orientation and negative values indicate a rightward orientation relative to a horizontal axis in the walk area. Yaw saccades are indicated by the black arrows. **(B)** Angular velocities of the yaw orientations depicted in **(A)**. Positive values represent leftward turning velocities while negative values symbolize turning velocities to the right. Exemplary yaw saccades are indicated by black arrows. **(C)** Average responses of left HSE-cells (resting potential subtracted) to behaviorally generated optic flow. Note that left HSE responses are approximated by recording from right HSE cells stimulated with mirror-symmetrical movies. In the upper row, responses to the original and stride-removed stimulus movies under the respective visual condition are shown. The lower row illustrates responses to original movies and to movies with modified texture properties. **(D)** Azimuthal position of the bar relative to the head yaw orientation of the fly. The dotted lines represent the motion direction of the bar or background texture, respectively. Note that the angle subtended by the fixation point and an arbitrary other point on the screen is given by the arctangent of the ratio between the metric distance between these two points and the distance of the observer from the point of fixation. Hence, the angular retinal position of the bar’s center converges to the angular position of one of its edges, if the fly fixates and approaches the other edge. **(E)** Average responses of right HSE-cells to behaviorally generated optic flow. As in **(C)**, the upper row, illustrates responses to the original and stride-removed stimulus movies while the lower row illustrates the responses to the original and modified movies. Sample size: stationary condition: 8 flies, except stride-removed left: 5 flies and stride-removed right: 7 flies; moving bar condition: 7 flies; moving background condition: 7 flies except stride-removed left: 3 Flies.

We assessed the neural response components evoked by the bar and the background, respectively. To this aim, the responses to the above described original ego-perspective movies were compared to targeted modifications of them. Two such modifications were employed: (1) From the original ego-perspective movie the bar texture was removed and the arising space filled in with background squares, leaving the background texture as the only structure in the environment (“background only”); and (2) the background texture was removed from the ego-perspective movies leaving the bar texture as the only environmental structure (“bar only”). Stride-removed stimulus: to reduce the effects of the stride-induced gaze shifts on the neuronal activity and, thus, to assess their functional significance, we smoothed the head yaw orientation trace by low-pass filtering (Butterworth filter 2nd degree, relative cut-off frequency: 0.45). We verified for this “stride-removed stimulus” that changes in the mean head yaw orientation were minimal (Figure 2, yaw data). Nevertheless, the filtering affected saccadic yaw turns and their velocities. However, as we do not focus on saccadic responses in this report, this modification does not have any impact on our conclusions. Becasue we filtered only head orientation data, the head position was not altered by the filter process.

To approximate the response of the HSE neuron contralateral to the recording side (i.e., in the left brain hemisphere), we presented mirror-symmetrical versions of the original stimulus movies while recording the activity of the right HSE cell. Consequently, tethered flies were confronted with a stimulation protocol of 24 movies in total, consisting of eight stimulus movies for each of the three visual conditions (stimulus movies: (1) original; (2) bar only; (3) background only; (4) stride-removed; and (5–8) mirrored version of (1–4)). Movies were presented in pseudorandom order with interstimulus intervals (ISI) of 3 s. During the first 2.5 s of the ISI, LEDs were operated at the average luminance level of the preceding movie, and the recorded cell’s resting potential was measured. In the last 0.5 s of the ISI, the LEDs faded to the first image of the upcoming movie.

### Data analysis

Data analysis was based on 3–8 HSE cells recorded in the right brain hemisphere. Therefore, cells recorded with the original movies will be termed “right HSE cells” whereas recordings with the mirror-symmetrical stimulus movies, simulating recordings from the left HSE cell, will be termed “left HSE-cells” in text sections and “simulated left HSE-cells” in figures. Data analysis was conducted with MATLAB R2011b. Since our analyses mainly focused on membrane potential changes around the resting potential, the recorded membrane potentials were subsequently set to zero for data analysis and presentation, by subtracting the measured resting potential from the overall response. The resting potential was measured for 1.5 s before the stimulus sequence started, while presenting the average brightness. Only recordings with stable resting potentials more negative than −35 mV were included into analysis. Average responses were calculated from individual responses to 2–12 stimulus repetitions per stimulus movie. To smooth out small action potentials of variable size, so-called spikelets, frequently superimposed on the graded membrane potential changes of HSE (Hausen, 1982a), responses were filtered with a Gaussian filter (filter width: 41 data points = 5 ms; sigma: 12).

#### Average stride-induced response

To draw conclusions on the stride-induced component of the HSE response, we took into account the stride data of the approach walks on which the presented motion stimuli were based. Strides are defined as the period in which all six legs performed a step. We estimated the stride timing from the video footage of the approaching fly by noting when the left mid leg touched the ground (Kress and Egelhaaf, 2014).

#### Receptive field coverage

By comparing the bar’s extent in the field of view with the size and the position of HSE’s receptive field, we calculated the bar’s relative receptive field coverage. According to previous findings, we roughly approximated the receptive field of HSE to range in azimuth from about 20° contralateral to 173° ipsilateral (Hausen, 1982b; Krapp et al., 2001). The extent of the receptive field in elevation was not included in this approximation.

#### Retinal expansion velocity and corresponding neural responses

The azimuthal expansion velocity of the bar was calculated as the difference between the retinal velocities of the bar’s right and left edge throughout the approach walk. The vertical velocity components were disregarded due to the horizontal motion preference of the analyzed HSE cells and their restricted sensitivity in elevation (Hausen, 1982b; Krapp et al., 2001). To compare responses of the right and the left HSE cell, we took the response asymmetry for preferred and null direction motion into account (stronger depolarizations than hyperpolarization) and normalized the responses separately for depolarizations and hyperpolarizations from the resting potential.

## Results

We analyzed the responses of a particular LPTC, the HSE cell, to combinations of self-induced image motion and external motion cues. The self-induced image sequences were obtained from reconstructing what walking blowflies had seen while fixating and approaching a vertical bar. These image sequences, thus, reflected the consequences of both goal-directed changes in the heading direction as well as stride-induced gaze shifts. HSE cells are depolarized by front-to-back motion and hyperpolarized by back-to-front motion (Hausen, 1982a,b) and have been concluded to be fundamental in mediating gaze stabilization, but also in the acquisition of spatial and object-related visual information (reviews: Egelhaaf, 2006; Taylor and Krapp, 2008; Borst et al., 2010; Egelhaaf et al., 2012).

### Responses to original and modified stimulus movies

HSE responses to optic flow experienced during walking were not only shaped by goal-directed changes in walking angle and forward translation, but to a large extent by periodic stride-coupled gaze shifts. Regular left and right gaze shifts coupled to the stride cycle elicited strong de- and hyperpolarizations of the membrane potential (Figure 2). Gaze shifts to the left depolarized the right HSE cell while hyperpolarizing the left HSE cell. Accordingly, stride-coupled gaze shifts to the right hyperpolarized the right HSE cell while depolarizing the left one. Periodical modulations of the cells’ activity were prominent during walking phases that were fairly straight apart from the stride-coupled fluctuations (Figure 2, red shaded area) as well as during phases of object-oriented changes in walking direction. In contrast, the changes in walking direction themselves had a less obvious effect on the HSE responses.

The impact of stride-induced image motion became especially obvious when comparing the responses to the original movies with the responses to stride-removed movies (Figures 2Ci,Ei). Stride-removed stimulus movies approximated the visual input perceived by walking flies without stride-coupled gaze shifts. When comparing the head yaw velocity for this condition with the respective responses, it was evident that HSE responses were then mainly shaped by goal-driven changes in walking direction (compare green curves in Figures 2B,Ci). Basically the same conclusion can be drawn with respect to the contralateral HSE-cell (compare turquois curves in Figures 2B,Ei).

To assess the neural response components evoked by environmental features like the bar and the background, we manipulated the stimulus movies in two ways. In the first manipulation, we removed the bar from the original movie, leaving the background texture as the only structure in the environment (background only). In the second manipulation the background texture was removed from the original movie, leaving the bar as the only environmental structure (bar only).

Removing either the bar or the background revealed that these features affected the response amplitude surprisingly little compared to the stride-induced image displacements (compare* red, blue and black curves* in Figures 2Cii-iii,Eii-iii). Response components evoked by object or background motion were dominated by the much larger response components resulting from the fly’s self-motion, in particular from the stride-coupled image shifts. Nevertheless, responses to external cues were visible in a direct comparison of the responses to the original stimulus with those to the modified stimulus movies. Under the stationary condition, i.e., when both bar and background did not move, response modulations were slightly reduced in the responses to the background only movie (Figures 2Ciii,Eiii: red curve), indicating the object-induced response component (Figure 2C–E left graphs). Under the moving bar condition, i.e., when the bar moved in front of the stationary background, responses to stimuli in which the bar was present (Figures 2Cii,Eii: black and blue curve) showed stronger hyperpolarizations to bar motion in null direction (Figures 2C,E middle graphs). In the moving background condition, i.e., when the background moved while the bar was stationary, the cells’ responses were shifted to slightly more depolarized or hyperpolarized values according to the direction of background motion. The responses to the stimulus movie with the object missing were more depolarized for background motion in preferred direction, while the response modulations were reduced in the contralateral cell for which the background was moving in null direction (Figures 2Ciii,Eiii, right graphs). This response difference was likely to be a consequence of the fact that in the background-only situation those areas of the visual field that were normally stimulated by the object were now covered by the moving background. Intriguingly, the most pronounced responses to external motion cues were apparent during stop phases, during which no stride-induced image shifts occurred (Figure 2, blue shaded areas indicating bar position related motion effects). This observation once more underlines the strong impact of stride-induced image shifts on motion signaling of HSE during walking.

As the fly approached the bar, its retinal image covered an increasingly larger extent of the receptive field of HSE-cells (Figure 2D). Consequently, the responses to the manipulated movies with the bar removed, tended to differ the more from the original response the closer the animal was to its goal. This increasing difference indicated that the object-induced response components got larger. This tendency became apparent in the responses of the respective cells when the receptive fields were covered by the bar during the end phases of the stimulus movies when the fly was directly in front of the bar (Figures 2Ciii,Eiii right most response parts).

### Average stride-induced response

To quantify the average stride-induced neuronal response components, we scrutinized the cells’ membrane potential changes around stride cycles. As the average stride cycle had a duration of 85 ms, we took the stride-triggered average responses 40 ms before and 40 ms after the reference point in the stride cycle (i.e., the touchdown of the left mid leg). Data on strides directly before and after stop-phases were ignored due to irregular reorientation saccades and irregular acceleration as well as deceleration effects on the head orientation (Kress and Egelhaaf, 2014).

**Figure 3:**
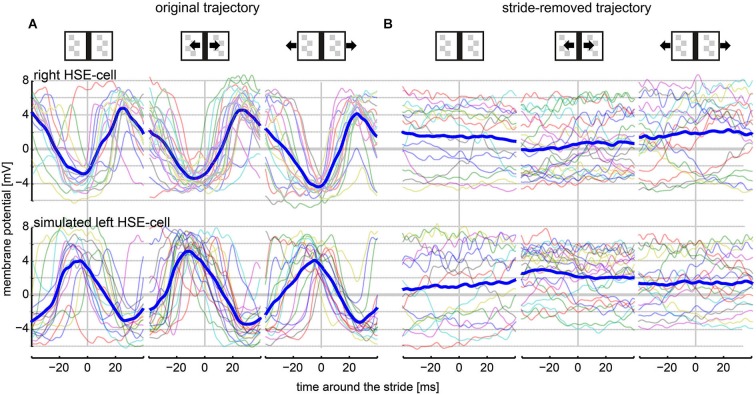
**HSE responses within a stride cycle (80 ms)**. Only responses to strides during continuous walking were included into this analysis (see Section Materials and Methods: Average Stride-Induced Response). The vertical line at the 0-ms mark indicates the stride cycle start/end as defined as the touchdown of the left midleg on the ground surface. **(A)** Stride-coupled responses of the right and the left HSE when stimulated with the original movie. Average responses (thick, dark blue lines) are shown together with the respective individual stride-coupled responses (semi-transparent, colored lines). **(B)** HSE responses within the stride cycle when stimulated with the stride-removed movie. Analyzed amount of strides: stationary condition: 15 strides; moving bar condition: 22 strides; moving background condition: 17 strides.

Stride-induced image shifts shaped the response of HSE cells strongly (Figure 3A). Stride-triggered average responses were similar across conditions with and without external motion and had peak-to-peak modulations of about 8 mV. Evoked depolarizations were under all conditions larger than hyperpolarizations. The right HSE membrane potential hyperpolarized directly before the end of a stride cycle and depolarized again thereafter. The membrane potential of the left HSE cells showed an inverted stride-induced response pattern.

The impact of strides on the neuronal response was particularly obvious in a direct comparison with the responses to the stride-removed stimulus movies. Average responses for the same intervals appeared flat in the latter condition (Figure 3B). On the one hand, the reduction in response modulations was a consequence of the missing stride-induced retinal image motion. On the other hand, response components evoked by object-induced changes in walking direction and, accordingly, retinal object position had in general a slower time course than a single stride cycle. Exceptions were responses evoked by saccadic turns (examples indicated by black arrows in Figure 2B). Interestingly, the slightly depolarized membrane potential in both the right and the left HSE cell indicated that the cells did not only respond to rotational image motion. If this were the case, the polarity of responses would be inverted in right and left cells (Figure 3B, compare upper and lower row). Thus, the simultaneous depolarization of the HSE-cells in both hemispheres was a consequence of forward translation, since only this type of self-motion induces preferred direction motion simultaneously in both cells.

### Object and background effects on HSE responses

**Figure 4:**
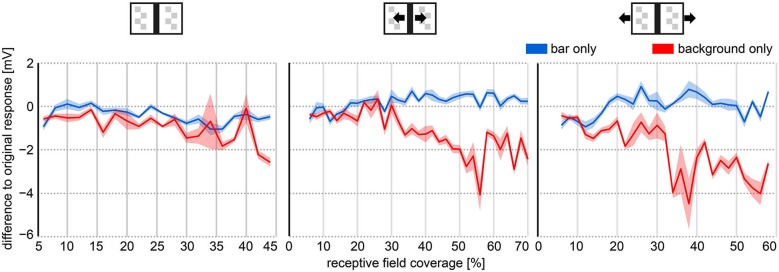
**Object and background effects on HSE responses as a function of the receptive field coverage of the bar**. The object and background effects were determined as the response differences between the average response to the modified texture stimulus movies (Figures 2C,E red and blue curve) and the average response to the original stimulus movie (Figures 2C,E; black curve). Receptive field coverage was binned in 2° bins. The response differences were averaged within these bins. Receptive fields of HSE cells were roughly approximated to range from 20° contralaterally to 172° ipsilaterally in azimuth. Right and left HSE responses were pooled. Thick lines represent average response difference, shaded areas represent the SEM.

As described above, HSE responses to image sequences as experienced by freely walking flies were modulated mainly by the image motion induced by stride-coupled gaze shifts. However, subtle response components evoked by the object and the background during goal-directed behavior were apparent as well. We found clear bar and texture related response components especially in the last phase of the fly’s approach to the bar. The strength of bar-related response components appeared to be coupled to the bar’s retinal position, the direction of motion of both bar and background, and the extent to which the bar covered the receptive fields of the right and left HSE cells. For quantitative analysis, we estimated the bar’s relative coverage of the receptive field of each of the two HSE cells (see Section Materials and Methods: Receptive Field Coverage) and related this parameter to the corresponding response difference between responses to the original movie and the modified versions of the movie (i.e., the background only and bar only movie). We then averaged the response differences (jointly for the right and left cell) as a function of the receptive field coverage for 2° bins (Figure 4).

Irrespective of the external motion condition, the bar had a larger impact on the neuron’s response than the background (wide-field stimulus). Nearly irrespective of the bar’s coverage of the cell’s receptive field, responses to the “background only” movies (without bar) differed more from the responses to the original movie than responses to “bar only” movies (without background) (Figure 4). Under the stationary condition, the response modulations to stimulus movies without bar were smaller resulting in a negative response difference. Under the moving bar condition, the negative response difference was the consequence of large hyperpolarizations evoked by the bar moving in the cell’s null direction. Under the moving background condition, the background depolarized the cell much more when the bar was missing (background only) and did not cover large parts of the receptive field of the cell. Therefore, also the response difference between original and background only stimulus movie was negative (compare red and black curves in Figures 2Ciii,Eiii).

The effect of the bar on the neuronal response increased with the receptive field coverage by the bar. This can be seen by the increasing deviations of the responses to the background only stimulus from the responses to the original stimulus (Figure 4, red line). This was also true for the other stimulus conditions, although the response deviations from the original situation were larger for the moving bar condition than for the stationary bar condition and even more for the moving background condition. These results indicate that HSE responses contain, in addition to stride-coupled self-motion information, information about stationary and moving structures in the environment. As expected from its receptive field properties, external wide-field motion affected HSE responses more than the movement of an object.

### Bilateral response sum as proxy for bar expansion velocity

In contrast to flight with its relatively long intersaccadic, virtually pure translational movements, distance estimation might be impaired in walking flies by the ongoing stride-coupled image rotations that superimpose translational image motion even during otherwise straight walking phases (Kress and Egelhaaf, 2012). However, distance estimation might be possible despite the stride-coupled image displacements on the basis of a purely visual mechanism, i.e., by extracting the bar’s expansion velocity from the perceived optic flow. Retinal bar expansion velocity increases with increasing nearness of the fly to the bar and might thus be extracted by comparing the retinal velocity of the bar’s right and left edges (Kress and Egelhaaf, 2014). Here, we tested to what extent this simple computation might be approximated by comparing the responses of the ipsi- and contralateral HSE cells, which are known to represent nearness information about objects during intersaccadic flight phases (Kern et al., 2005; Karmeier et al., 2006; Liang et al., 2008, 2012).

**Figure 5:**
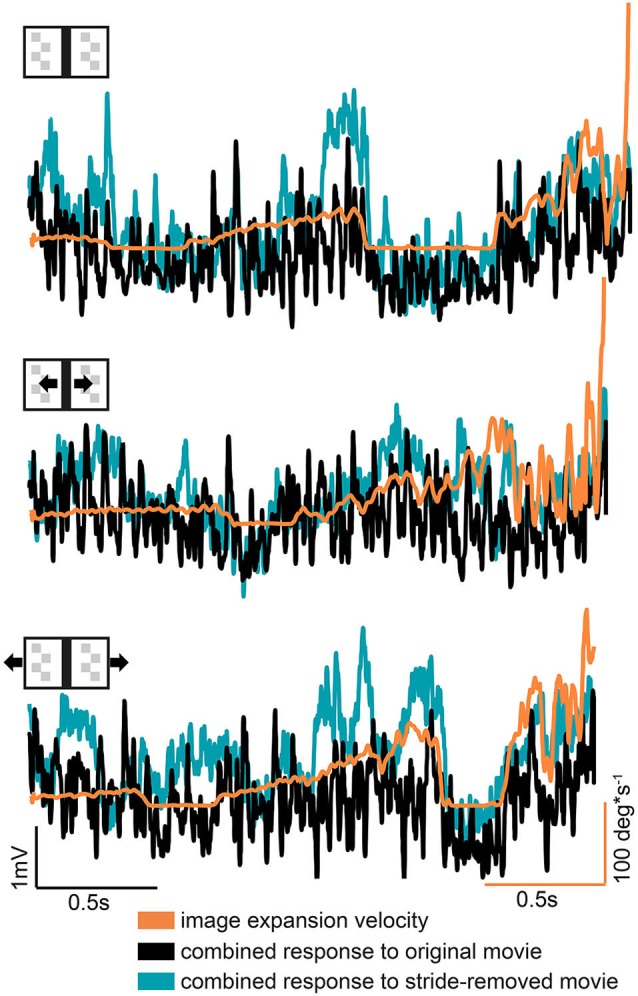
**Combined bilateral response compared to the bar’s expansion velocity**. The combined response was obtained by summing the responses of both hemispheres after they were normalized separately for motion in the preferred and null direction motion. Note that response and velocity curves have different scales and units.

During a frontal approach towards the bar the right HSE cell can be expected to be driven to some extent by the moving right edge of the bar, whereas the left HSE cell is assumed to be driven by the moving left edge. To assess to what extent a measure of the bar’s expansion velocity can be derived from the neural responses we, therefore, summated the normalized responses of both cell. Compared to the original stimulus movie, the stride-removed stimulus movie evoked slightly stronger response sums and a somewhat stronger correlation with the bar’s expansion velocity: we found a weak correlation between the retinal expansion velocity of the bar with the bilateral response sum obtained for both the original and the stride-removed stimulus movies (correlation coefficients: all stationary condition: *R*_original_ = 0.38; *R*_stride-removed_ = 0.43; moving bar condition: *R*_original_ = 0.16; *R*_stride-removed_ = 0.4; moving background condition: *R*_original_ = 0.32; *R*_stride-removed_ = 0.43). However, as indicated by the low correlation coefficients this dependency is weak, because large response sums occurred not only at high expansion velocities, but also in sections of the response traces where image expansion velocity was relatively small (Figure 5 left side). This result might be partly due to the different sensitivity of the HSE cell in different parts of its receptive field (Hausen, 1982b; Krapp et al., 2001), the nonlinear velocity tuning of HSE and the fact that its responses depend as well on pattern contrast and texture (review: Egelhaaf et al., 2012).

Based on these findings, we conclude that the comparison of the HSE responses appear to be insufficient to serve as the only input for a visual mechanism to compute image expansion and thereby to obtain distance information. Therefore, other mechanisms, potentially non visual ones, are suggested to cope with the visual consequneces of stride-coupled rotational image shifts.

## Discussion

We conducted intracellular recordings from HSE cells in the visual system of the blowfly. These cells are motion sensitive wide-field neurons. Being output neurons of the motion vision pathway and receiving input from a retinotopically arranged array of local motion sensitive elements as well as from wide-field cells of the contralateral optic lobe (reviews: Egelhaaf, 2006; Borst et al., 2010), they have been concluded to play a role in optomotor behavior as well as in self-motion and spatial information processing (review: Egelhaaf et al., 2012). Our aim was to assess how self-produced periodic image shifts, resulting from leg movements, affect the activity of these visual interneurons in a goal-directed paradigm. The experiments were done in immobilized animals. The visual stimuli were based on previous behavioral experiments, in which walking flies approached a bar, and represent the flies’ complex spatio-temporal visual input experienced during this task (Kress and Egelhaaf, 2014). We presented stimuli where either the bar or the background moved, and we compared responses to stimuli that contain the stride-coupled rotational image shifts with the responses to stimuli in which we removed this component to a large extent. In this way we assessed how self-produced image motions as well as external motion affect the neural representation of information about the environment.

### The impact of locomotion-based image shifts

We found that stride-coupled gaze shifts dominate the responses of HSE cells. The membrane potential was modulated periodically by the stride-coupled image displacements at a frequency of about 12 Hz. This was also the case in walking phases that were straight apart from the stride-coupled fluctuations. Consequently, goal-driven changes in the walking direction were less prominent in the cell’s response.

Nevertheless, environmental features were also represented in the cell’s response. The more the fly approached the black bar, the more did the bar cover the cell’s receptive field and the larger was the bar’s expansion velocity on the eyes. As a consequence, the response component evoked by the bar increased. Throughout the approach, HSE responses were affected more strongly by the bar than by the background. This characteristic might result from the high contrast of the bar and from the coverage of large parts of the receptive field towards the end of the approach. Moreover, translation-based image motion is an additional feature affecting responses of HSE cells (Liang et al., 2008, 2011, 2012).

### Potential consequences of the behavioral state on HSE responses

Our electrophysiological recordings were done in tethered flies that were confronted with reconstructed image motion of walking conspecifics. Recent findings demonstrate that visual motion processing is affected by the current activity state of the animal (Longden and Krapp, 2009; Chiappe et al., 2010; Maimon et al., 2010; Rosner et al., 2010; Jung et al., 2011; Rien et al., 2012). Therefore, even when using naturalistic visual input sequences neuronal responses of a tethered fly may not reflect the neuronal activity present in a fly when walking. However, it is likely to assume that HSE responses to both, stride-coupled and externally-caused image motion should be affected similarly by the behavioral state. Therefore, we believe that these effects might affect our results mainly quantitatively but not qualitatively. If stride-coupled and external image motion were differentially affected, one would rather expect a stronger state-dependent enhancement of the responses to stride-induced motion than of the responses to external motion. This expectation is based on the finding that locomotor activity induced a shift of neuronal tuning of LPTCs towards higher velocities (Chiappe et al., 2010; Jung et al., 2011). For the paradigm used in the present study high velocities are more prevalent during stride-coupled image shifts than during external motion. However, state-dependent tuning shifts were not found in all studies (Suver et al., 2012). Moreover, in another study it was shown that neuronal responses to naturalistic optic flow (reconstructed from flight data) were not fundamentally altered by octopamine, a neuromodulator that mediates the state dependence (Rien et al., 2013).

### Extraction of environmental information

In a previous account, we proposed that the retinal expansion velocity of the bar might be extracted from the overall retinal image flow even in the presence of the stride-coupled rotational component (Kress and Egelhaaf, 2014). This is possible, at least in principle, solely based on visual information by subtracting the velocities of the bar’s two edges. Therefore, we asked how well this simple computation might be approximated by combining the antagonistic motion responses of the left and right HSE cells. Although we found a weak correlation between the combined response and the retinal expansion velocity of the bar, the combined signal does not provide unambiguous distance information. Possible reasons are that HSE responses represent image velocity nonlinearly, that they are affected by the pattern properties (review: Egelhaaf et al., 2012) and that the impact of the edges on the neural responses varied according to the spatial sensitivity profile of the cell’s receptive field (Hausen, 1982b; Krapp et al., 2001).

There might be other ways of interactions within the visual system to cope with the consequences of the stride-coupled image shifts. FD1 cells, another type of motion sensitive wide-field neuron, might be less affected by stride-coupled retinal image shifts (Kimmerle and Egelhaaf, 2000a; Liang et al., 2012). FD1 cells are most sensitive to front-to-back motion of an object in the ipsilateral hemisphere and are inhibited by wide-field motion in both, the ipsi- as well as the contralateral hemisphere (Egelhaaf, 1985). This inhibition is mediated by horizontal motion sensitive CH cells (Warzecha et al., 1993). Since CH cells respond, in contrast to HSE-cells (Hausen, 1982b; Kern et al., 2005; Hennig et al., 2011), only weakly during translatory motion (Eckert and Dvorak, 1983; Egelhaaf et al., 1993; Farrow et al., 2003; Hennig et al., 2011), their responses can be hypothesized to be driven much more strongly by the stride-coupled image rotations than by the translatory optic flow component which results from approaching the object (Hennig and Egelhaaf, 2012). Consequently, inhibitory input from CH cells might reduce FD1’s overall stride-coupled response components and, thus, might accentuate its responses related to the object (see also Kimmerle and Egelhaaf, 2000b).

However, there might be also non-visual mechanisms that could deal with the consequences of stride-coupled image shifts. Flies might use information provided by other sensory modalities to eliminate rotation-based visual responses that superimpose distance dependent translational responses. Mechanosensory feedback from the haltere-system (Sandeman, 1980a,b; Nalbach and Hengstenberg, 1984) or the pedal system (Horn, 1982) might be used not only to control compensatory head movements but also to modulate the visual responses to stride-coupled rotations. Because we recorded the neuronal activity of tethered animals such multimodal interactions were prevented and, therefore, their putative effects on the responses of HSE cells could not be observed. However, since we do not have much evidence for a mechanosenory input at the level of HSE cells, that are thought to be mainly visual interneurons, multimodal interactions destined to reduce the consequences of stride-coupled rotational image motion are more likely to be affected, if they exist at all, at more downstream processing stages.

Another possibility to cope with the consequences of rotational optic flow on distance estimation might be the involvement of an efference copy that generates a rotation-proportional output and might originate in the motor control system. Evidence for the role of such a mechanism in fly optomotor behavior has been provided by the seminal study of Holst and Mittelstaedt (1950) and later by Heisenberg and Wolf (1988). Such an efference copy is a representation of a motor command and might modulate the responses of the optomotor system to stride-coupled rotations. If such a mechanism plays a role in modifying the output of the visual motion pathway, it may operate at all processing stages where stride coupled rotational signals are represented and superimpose the information about the environment also present in the neural signals. In principle, this might be the level of LPTCs, but also more downstream processing stages, This issue has not yet been resolved, mainly because most electrophysiological studies were performed, so far, for methodological reasons on immobilized animals. Moreover, the few studies comparing at the level of LPTCs the motion responses of inactive and tethered flying or walking flies, did not address explictly the potential influence of an efference copy of behavioral commands on the neural responses (Chiappe et al., 2010; Maimon et al., 2010; Jung et al., 2011; Longden et al., 2014). Just one recent study indicates, although it did not investigate this issue systematically, that motion responses of HSE cells might be affected during tethered flight by an intended saccadic turn. This input could be observed even under conditions where the turn could not be physically executed (open loop conditions) and, thus, did not have a direct effect on the visual input (see Figure 1C in Schnell et al., 2014). It will be one issue of forthcoming studies to find out whether such effects can also be observed in walking flies and whether they are stride-coupled even if the animal walks in an overall straight way.

Although an efference copy as well as reafferences from mechanosensors might well be utilized to reduce the impact of self-induced image rotations in visual interneurons, both mechanisms cannot exactly predict the strength of visual responses to self-rotations. The responses of HSE cells like those of other fly motion sensitive interneurons do not only depend on stride-coupled retinal velocities, but, in addition, on the spatial frequency content and local contrast of the stimulus pattern (Borst and Egelhaaf, 1989; Egelhaaf and Borst, 1989; Warzecha and Egelhaaf, 2000; Straw et al., 2008). Hence the response strength of such neurons may vary a lot even for a given velocity of image rotational depending on the textural properties of the image.

The impact of stride-coupled rotational image displacements for spatial vision is currently being investigated in our lab with tethered flies walking on a trackball in virtual reality under both open- and closed-loop conditions. It should be noted, however, that in animals where the body and head orientation is fixed in space as a consequence of the tether, stride-coupled gaze shifts cannot be observed without further tricks. This was, most likely, the major reason that the pronounced stide-coupled gaze shifts were discovered only recently (Kress and Egelhaaf, 2012), despite the large number of studies on tethered walking flies.

### Why are stride-coupled body yaw turns not compensated?

So far, we have discussed by what mechanisms flies may cope with the consequences of stride-coupled retinal image shifts. However, one might also ask why such image shifts occur at all. Given our result that HSE cells are driven to a large extent by stride-coupled image rotations, it is surprising that walking blowflies do not compensate self-produced horizontal image shifts by counter-rotating their head (Kress and Egelhaaf, 2012, 2014). Several studies have shown that, in general, such optomotor reflexes exist in flies, thus reducing rotational retinal image slip and supporting visual information processing (Götz and Wenking, 1973; Götz, 1975; Srinivasan, 1977; Hengstenberg, 1984). However, most of these studies worked with flying or walking flies that were tethered at their thorax and had their head fixed to the thorax. Therefore, these studies primarily analyzed optomotor yaw responses of the body to horizontal motion. How the head is turned independently of the body to compensate for image shifts was not analyzed systematically except of few studies dealing during tethered flight with haltere-mediated head compensation as well as with head movements induced by object and visual wide-field motion, respectively (Nalbach and Hengstenberg, 1984; Fox and Frye, 2014). As body roll is largely compensated by visually induced compensatory head roll (Schwyn et al., 2011; review: Hengstenberg, 1984; van Hateren and Schilstra, 1999) and because HS cells project onto the neck-motor system (Strausfeld and Seyan, 1985; Milde and Strausfeld, 1986; Huston and Krapp, 2008, 2009; Haag et al., 2010; Wertz et al., 2012), it is generally assumed that freely walking blowflies perform also visually induced head yaw compensation. Our finding that this is not the case during unrestrained walking makes it necessary to analyze carefully the behavioral conditions under which compensatory head movements are generated.

## Conflict of interest statement

The authors declare that the research was conducted in the absence of any commercial or financial relationships that could be construed as a potential conflict of interest.

## Footnotes

^1^http://oss.sgi.com/projects/inventor/license.html
